# Joint analysis of D-dimer, N-terminal pro b-type natriuretic peptide, and cardiac troponin I on predicting acute pulmonary embolism relapse and mortality

**DOI:** 10.1038/s41598-021-94346-7

**Published:** 2021-07-21

**Authors:** Xiaoyu Liu, Liying Zheng, Jing Han, Lu Song, Hemei Geng, Yunqiu Liu

**Affiliations:** 1 Department of Respiratory and Critical Care Medicine, Kailuan General Hospital, 57 East Xinhua Rd, Tangshan, Hebei China; 2 Hospital Infection Management Division, Kailuan General Hospital, 57 East Xinhua Rd, Tangshan, Hebei China

**Keywords:** Respiratory tract diseases, Embolism

## Abstract

Previous studies on the adverse events of acute pulmonary embolism (APE) were mostly limited to single marker, and short follow-up duration, from hospitalization to up to 30 days. We aimed to predict the long-term prognosis of patients with APE by joint assessment of D-dimer, N-Terminal Pro-Brain Natriuretic Peptide (NT-ProBNP), and troponin I (cTnI). Newly diagnosed patients of APE from January 2011 to December 2015 were recruited from three hospitals. Medical information of the patients was collected retrospectively by reviewing medical records. Adverse events (APE recurrence and all-cause mortality) of all enrolled patients were followed up via telephone. D-dimer > 0.50 mg/L, NT-ProBNP > 500 pg/mL, and cTnI > 0.40 ng/mL were defined as the abnormal. Kaplan–Meier curve was used to compare the cumulative survival rate between patients with different numbers of abnormal markers. Cox proportional hazard regression model was used to further test the association between numbers of abnormal markers and long-term prognosis of patients with APE after adjusting for potential confounding. During follow-up, APE recurrence and all-cause mortality happened in 78 (30.1%) patients. The proportion of APE recurrence and death in one abnormal marker, two abnormal markers, and three abnormal markers groups were 7.69%, 28.21%, and 64.10% respectively. Patients with three abnormal markers had the lowest survival rate than those with one or two abnormal markers (Log-rank test, *P* < 0.001). After adjustment, patients with two or three abnormal markers had a significantly higher risk of the total adverse event compared to those with one abnormal marker. The hazard ratios (95% confidence interval) were 6.27 (3.24, 12.12) and 10.7 (4.1, 28.0), respectively. Separate analyses for APE recurrence and all-cause death found similar results. A joint test of abnormal D-dimer, NT-ProBNP, and cTnI in APE patients could better predict the long-term risk of APE recurrence and all-cause mortality.

## Introduction

Acute pulmonary embolism (APE) is one of the most frequent cardiovascular diseases which characterizes the blockage of pulmonary arterial bed by thromboemboli, usually a consequence of deep vein thrombosis (DVT)^1^. It is the most serious clinical outcome of venous thromboembolism (VTE), with a high mortality rate following myocardial infarction and cancer. Untreated APE could have a mortality rate as high as 30%, whereas diagnosis and treatment could lower the disease-attributed mortality rate to 2–8%, with the recurrence rate at 4–17%^2^. Comprehensive examinations and treatment are therefore essential for APE patients.

D-dimer, N-terminal pro b-type natriuretic peptide (NT-proBNP), and Cardiac troponin I (cTnI) are important biomarkers of the occurrence, development, and prognosis of APE. D-dimer is a biomarker for activation of coagulation and fibrinolysis. NT-proBNP is released due to increasing myocardial stretch and is positively associated with the severity of right ventricular dysfunction. cTnI specifically reflects myocardial injury. These biomarkers have also been reported for predicting clinical adverse events in APE patients^3–5^.

Current literature on the adverse events of APE was mostly limited to mortality in the short duration of hospitalization^6,7^, or up to 30 days^8,9^. A few studies suggested that these clinical tests may potentially be associated with long-term prognosis^10,11^. Because of the specificity limitation of any single biomarker^1^, combinations of these tests have been created and applied to improve risk stratification^12–16^. This study aimed to analyze the joint effect of D-dimer, NT-proBNP, and cTnI on predicting long-term adverse event risk on APE patients.

## Methods

### Study population

Physician retrospectively reviewed medical records of hospitalized APE patients in the North China University of Science and Technology Affiliated Hospital, Tangshan Workers’ Hospital, and Kailuan General Hospital during January 1st, 2011 and December 31st, 2015. This study was approved by the Review Board of the Kailuan General Hospital. All methods were performed in accordance with the relevant guidelines and regulations and were in accord with the Declaration of Helsinki. All participants provided written informed consent. Inclusion criteria were: (1) incident APE patient, (2) no history of chronic PE, and (3) aged over 18 years. Exclusion criteria were: patients who (1) received thrombolytic or anticoagulant therapy prior to hospitalization, (2) accompanied by thrombotic diseases, myocardial infarction, cerebrovascular diseases, hematologic diseases, infectious diseases, sepsis, liver diseases, or kidney diseases, or (3) had no D-dimer, NT-proBNP, and cTnI tests.

Due to the lack of the risk ratio of the combination of indicators in the literature, we used the sample size calculation for cohort studies:

$$n=\frac{{({z}_{\alpha }\sqrt{2\overline{pq} }+{z}_{\beta }\sqrt{{p}_{0}{q}_{0}+{p}_{1}{q}_{1}})}^{2}}{{({p}_{1}-{p}_{0})}^{2}}$$

According to the literature, the APE mortality rate for the individual mortalities are around 11.2% for D-dimer > 0.50 mg/L^17^, 20.0% for cTnI > 0.05 ng/ml^18^, and 16.8% for NT-Pro BNP > 600 pg/Ml^1^, respectively. Based on these values, when a single marker is abnormal, the maximum mortality of cTnI abnormality is estimated as 20.00%, therefore p_0_ = 20.0%. We estimated that when all three indicators are abnormal, the mortality rate of APE should be higher than 50%, therefore p_1_ = 50.0%. Using the formula with α = 0.05 (bilateral) and β = 0.10, the sample size calculation resulted in a single group of 51 cases, and a total of 102 cases. Considering a 20% potential risk of loss to follow follow-up, at least 128 cases were required.

APE was diagnosed according to the 2015 Chinese Expert Consensus on Diagnosis and Management of Acute Pulmonary Embolism^17^. Patients received and were diagnosed on at least one of the following examinations: computed tomographic pulmonary angiography, ventilation-perfusion scintigraphy, and pulmonary artery angiography.

The following data were retrieved from medical records. Personal characteristics included hospital, age, sex, smoking status (never, past, or current smoker), and alcohol consumption status (never, past, or current drinker). Clinical and physical characteristics included change in psychological status (presence of anxiety, tiring, sleepiness, fatigue, frustration, unconsciousness, fear, and agony, etc.), heart rate, systolic blood pressure (SBP), D-dimer, NT-proBNP, and cTnI levels, and blood oxygen saturation (SaO_2_; prior to oxygen supply). Treatment details included thrombolytic therapy, anticoagulant treatment, and interventional therapy. Accompanied diseases included cancer and chronic cardiovascular diseases. Data were de-identified during analyses.

### Classification

All three hospitals used the same methods for assessments. D-dimers were quantitatively determined by enzyme-linked immunosorbent assay (ELISA), and NT-Pro BNP and cTnI were quantitatively determined by electrochemiluminescence immunoassay (ECLIA). Detailed analytical methods were summarized in Supplemental Table 1. According to the Chinese expert consensus on the diagnosis and treatment of acute pulmonary embolism in 2015, D-dimer > 0.50 mg/L was classified as abnormal^19^. According to the 2011 scientific statement of the American Heart Association on the treatment of large and sub-large pulmonary embolism, deep vein thrombosis, and chronic thromboembolic pulmonary hypertension, NT-ProBNP > 500 pg/ml and cTnI > 0.40 ng/ml were considered as abnormal and these values were used as cut-off values in our study^20^. Patients were further categorized by the number of abnormalities met: 0, 1, 2, or 3.

### Follow-up

Included APE patients were followed up via telephone call in May 2016 for adverse events and prescription adherence after discharge. Adverse events included all-cause mortality and APE recurrence. Prescription adherence was considered satisfactory if patients: (1) followed prescriptions without altering the drugs or dosage, (2) had regular revisits with their doctors, and (3) refrained from smoke, alcohol, and warfarin inhibitors. Deaths reported during telephone interviews were confirmed through linkage to the vital office for exact time and cause. Rehospitalization cases reported were confirmed through medical records. Patients who survived without APE recurrence were censored for an adverse event.

### Statistical analysis

Data were recorded and managed in EpiData version 3.1, and analyzed in SAS version 9.3 9SAS Institute, Cary, USA) after de-identification. Chi-square test and Fisher’s exact test were used to compared baseline characteristics across baseline markers abnormalities. Kaplan–Meier survival curves categorized by the number of abnormalities were plotted and compared by log-rank test for total adverse event, and APE recurrence or all-cause mortality separately.

Cox proportional hazard model was used to analyze the association between baseline abnormality number and adverse events. Time to event or censor was calculated from the date of hospitalization to the date of follow-up. Covariates included age, sex, heart rate, SBP, smoking, alcohol consumption, change in psychological status, SaO_2_, disease history, treatment, and prescription adherence. Prescription adherence was collected from patients who were alive at the time of follow-up and adjusted only in the analysis for APE recurrence. The significance level was set as α = 0.04. All tests were two-sided.

## Results

A review of medical records identified 432 APE patients. Of them, 13 were excluded for receiving thrombolytic or anticoagulant therapy prior to hospitalization; 20 were lost to follow-up; 43 did not receive D-dimer test, 96 did not receive NT-proBNP test, and 1 did not receive cTnI test. A total of 259 APE patients were included in the analysis.

At baseline, the mean age was 61.4 + 13.8 years, and 132 (51.0%) were men. Baseline characteristics of the included participants are presented in Table 1. Seven did not have abnormal values for either D-dimer, NT-proBNP, or cTnI tests; 67 had single abnormality; 117 had 2 abnormalities (Group 2); 68 had 3 abnormalities (Group 3). Due to the small number of patients without abnormal values, they were categorized together with the single abnormality group (Group 1).

**Table 1:** Baseline characteristics of newly-diagnosed acute pulmonary embolism patients.

	Total (*n* = 259)	Single abnormal marker (*n* = 74)	Two abnormal markers (*n* = 117)	Three abnormal markers (*n* = 68)	*P*
Age, year	61.5 ± 13.8	59.5 ± 13.8	62.0 ± 12.9	62.6 ± 15.2	0.34
Men, n (%)	132 (51.0)	37 (50.0)	60 (51.3)	35 (51.5)	0.98
Heart rate, time/min	89.2 ± 17.5	86.2 ± 16.0	87.6 ± 16.9	95.3 ± 18.9	0.003
SBP (mmHg)	131 ± 20	134 ± 14	129 ± 22	130 ± 22	0.26
**Smoking (*n*, %)**					0.01
Never	178 (68.7)	55 (74.3)	82 (70.1)	41 (60.3)	
Past	28 (10.8)	4 (5.41)	9 (7.7)	15 (22.1)	
Current	53 (20.5)	15 (20.3)	26 (22.2)	12 (17.7)	
**Alcohol consumption (*n*, %)**					0.006
Never	210 (81.1)	68 (91.9)	95 (81.2)	47 (69.1)	
Past	6 (2.32)	0 (0.00)	2 (1.71)	4 (5.88)	
Current	43 (16.6)	6 (8.11)	20 (17.1)	17 (25.0)	
Psychological change (*n*, %)	122 (47.1)	25 (33.8)	60 (51.3)	37 (54.4)	0.02
SaO_2_ < 90% (*n*, %)	68 (26.3)	19 (25.7)	33 (28.2)	16 (23.5)	0.78
History of cancer (*n*, %)	39 (15.1)	9 (12.2)	21 (18.0)	9 (13.2)	0.49
History of chronic cardiovascular disease (*n*, %)	189 (73.0)	46 (62.2)	95 (81.2)	48 (70.6)	0.01
**Treatment (*n*, %)**					0.18
Anticoagulant treatment	169 (65.3)	56 (75.7)	72 (61.6)	41 (60.3)	
Thrombolytic therapy	42 (16.2)	6 (8.11)	23 (19.7)	13 (19.1)	
Interventional therapy	48 (18.5)	12 (16.2)	22 (18.8)	14 (20.6)	

SBP, systolic blood pressure; SaO_2_, blood oxygen saturation.

**Figure 1:**
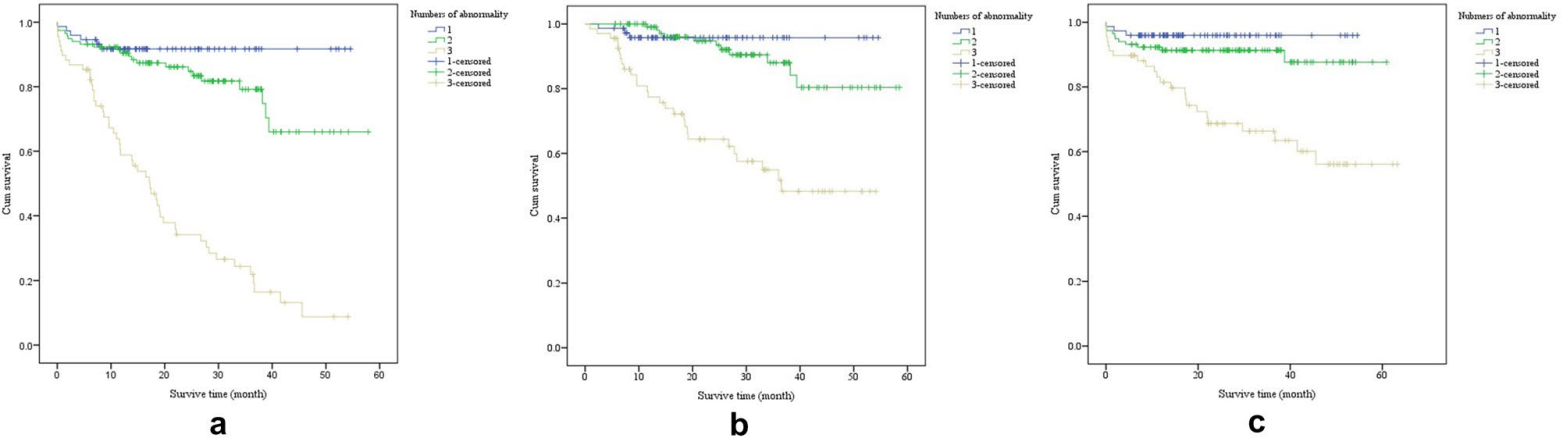
Kaplan–Meier survival curves for (**a**) total adverse event, (**b**) recurrence, and (**c**) all-cause mortality in acute pulmonary embolism patients categorized by number of abnormalities (log rank test *p* < 0.001 for all).

The median survival time to the total adverse event was 45.6 months. Group 3 had the shortest median survival time of 17.3 months, and a significantly lower survival rate than the other two groups (*p* < 0.001, Fig. 1).

Compared to Group 1, Group 3 had a significantly higher hazard ratio (HR) of 10.7 (95% confidence interval [CI]: 4.08 to 28.06) and 6.27 (95% CI: 3.24, 12.12, Table 2), compared to Groups 1 and 2, respectively, for adverse events. A significantly higher risk persisted in Group 3 after separating the outcomes. For APE recurrence, the HRs (95% CIs) compared to Groups 1 and 2 were 8.78 (2.49, 30.91) and 5.06 (2.18, 11.83); for all-cause mortality, HRs (95% CIs) compared to Groups 1 and 2 were 7.88 (1.70, 36.54) and 4.98 (1.72, 14.48), respectively (Table 2). No significant difference was found between Groups 1 and 2 (*p* > 0.05 for all outcomes, Table 2). Comparison between individual markers was presented in Supplemental Table 2.

**Table 2:** Association between number of abnormal markers (D-dimer, N-Terminal Pro-Brain Natriuretic Peptide, and troponin I) and acute pulmonary embolism prognosis.

	Single abnormal marker (*n* = 74)	Two abnormal markers (*n* = 117)	Three abnormal markers (*n* = 68)
**Adverse event**			
Event, n(%)	6 (7.69)	22 (28.2)	50 (64.1)
Model 1	1 (reference)	1.71 (0.61, 4.77)	10.7 (4.08, 28.0)
P		0.31	< 0.001
Model 2	0.59 (0.21, 1.64)	1 (reference)	6.27 (3.24, 12.1)
P	0.31		< 0.001
**APE recurrence**			
Event, n(%)	3 (7.32)	11 (26.8)	27 (65.9)
Model 1	1 (reference)	1.73 (0.44, 6.76)	8.78 (2.49, 30.9)
P		0.43	< 0.001
Model 2	0.58 (0.15, 2.25)	1 (reference)	5.06 (2.18, 11.8)
P	0.43		< 0.001
**All-cause mortality**			
Event, n(%)	3 (8.11)	11 (29.7)	23 (62.2)
Model 1	1 (reference)	1.59 (0.32, 7.93)	7.88 (1.70, 36.5)
P		0.58	0.008
Model 2	0.59 (0.12, 2.99)	1 (reference)	4.98 (1.72, 14.4)
p	0.53		0.003

Values are hazard ratio (95% confidence interval). Model 1 used Group 1 (single abnormal marker) as reference, while Model 2 used Group 2 (Two abnormal markers) as reference. Models adjusted for age, sex, heart rate, SBP, smoking, alcohol consumption, change in psychological status, SaO_2_, disease history, treatment, and prescription adherence (for APE recurrence). Adverse event included both APE recurrence and all-cause mortality.

## Discussion

This study found that joint test of D-dimer, NT-proBNP, and cTnI during hospitalization was able to predict long-term adverse events after discharge. Patients with abnormal values in all 3 markers had 11.3 and 6.4 times of risk of patients with one or two abnormalities.

Compared to patients with a single abnormality, a higher risk of adverse events in patients with two markers of abnormalities was not found in this study. This finding did not support previous studies on the joint effect of two tests. One study reported that APE patients with abnormal NT-proBNP and cTnI levels had a 30% mortality rate, while those with a single abnormal NT-proBNP only had a 3.7% mortality rate^18^. Similarly, another study found that APE patients with NT-proBNP ≥ 600 ng/L and cTnT ≥ 0.07 mg/L had a 33% mortality rate at 40 days after discharge^12^. Several reasons may explain the difference. First, due to the rapid progress of APE, there may be variability in three biomarkers^2^. Second, these studies were limited in small sample size which limited the ability to test the difference between groups^12,18^. Third, these biomarkers might have different prognostic values in predicting short-term versus long-term adverse event risk. Large prospective cohort studies are warranted for elucidating the association.

Studies have shown that biomarkers such as D-dimer, NT-proBNP, and cTnI are related to pulmonary artery obstruction. Pulmonary circulatory disturbances caused by APE can increase pulmonary artery pressure, leading to myocardial damage or right ventricular dysfunction^21^. D-dimer is a soluble degradation product of cross-linked fibrin under the action of the fibrinolytic system. Its level reflects the generation of thrombin and the activity of plasmin and has high clinical sensitivity for the diagnosis of APE^22^. NT-proBNP is mainly synthesized and secreted by the ventricular myocytes of the heart. When acute pulmonary embolism occurs, it will reduce the effective blood flow of the pulmonary vascular bed, increase the pressure of the pulmonary artery, increase the tension of the right ventricle, and cause right heart dysfunction^23^. As a specific marker of myocardial injury, a high cTnI level indicates impaired right ventricular function, which is more likely to cause right heart failure or cardiogenic shock in patients^24^. Together, these three markers suggest strong potential in predicting the long-term prognosis of individuals with APE. The survival plot in our study indicated that during the follow-up of around 4 years, the all-cause mortality rate kept at a high level (Fig. 1). Moreover, the current consensus on APE also focused on short-term adverse events, therefore the recommendations might not be able to effectively predict long-term outcomes. The joint analysis of 3 frequently used biomarkers might be of clinical significance for identifying high-risk patients. For patients with abnormal values of D-dimer, NT-proBNP, and cTnI together, special treatment and frequent revisit might be warranted to lower the risk for long-term recurrence and mortality.

Limitations of this study should be noted. First, due to the limited sample size, cause-specific deaths were not analyzed separately. The association between joint tests of D-dimer, NT-proBNP, and cTnI and APE-attributed mortality remained unclear. Second, 173 patients were excluded due to missing information or lost to follow-up. However, we compared the baseline characteristics of included versus the total identified participant and found no significant difference. Despite these limitations, the quality of our study was well controlled. Medical records of patients were reviewed by two physicians, where uncertain cases were decided by a third physician. Recurrence and mortality information reported during telephone interviews were confirmed by review of records or certificates. Various confounding factors were adjusted in our model.

To conclude, the presence of D-dimer, NT-proBNP, and cTnI abnormalities together in APE patients was associated with a higher risk of long-term recurrence and mortality compared to less or no abnormalities. Special care and follow-up might be important for these patients.

## Supplementary Information

Supplementary Information.

## Data Availability

Data would be made available from the corresponding authors upon reasonable request and approval.
