# Supplementary material for: Joint analysis of D-dimer, N-terminal pro b-type natriuretic peptide, and cardiac troponin I on predicting acute pulmonary embolism relapse and mortality
Source: Sci Rep. 2021 Jul 21;11:14909. doi: 10.1038/s41598-021-94346-7 (PMC8295248; doi:10.1038/s41598-021-94346-7)
Supplement: Supplementary file 1 — Supplementary Information. [file 41598_2021_94346_MOESM1_ESM.pdf]

Supplemental file for "Joint analysis of D-dimer, N-terminal pro b-type natriuretic peptide, and Cardiac troponin I on predicting acute pulmonary embolism relapse and mortality"

Xiaoyu Liu M*, Liying Zheng MD*, Jing Han MD, Lu Song MD, Hemei Geng MD†, Yunqiu Liu MD

Supplemental Table 1. Equipment and relevant analytical methods in the three hospitals for D-dimer, N-Terminal Pro-Brain Natriuretic Peptide, and troponin I.

Methods	North China University of Science and Technology Affiliated Hospital	Tangshan Workers' Hospital	Kailuan General Hospital
D-dimer			
Analytical method	VIDAS D-Dimer ExclusionII (DEX2)	VIDAS D-Dimer ExclusionII (DEX2)	VIDAS D-Dimer ExclusionII (DEX2)
Equipment	Automatic immunoassay analyzer VIDAS30	Automatic immunoassay analyzer VIDAS30	Automatic immunoassay analyzer VIDAS30
Detection range	45ng/mL - 10000ng/mL (FEU)	45ng/mL - 10000ng/mL (FEU)	45ng/mL - 10000ng/mL (FEU)
Reference range	0-0.5mg/L	0-0.5mg/L	0-0.5mg/L
N-Terminal Pro-Brain Natriuretic Peptide			
Analytical method	VITROS Immunodiagnostic Products NT-proBNP Reagent Pack	VITROS Immunodiagnostic Products NT-proBNP Reagent Pack	VITROS Immunodiagnostic Products NT-proBNP Reagent Pack
Equipment	Automatic analyzer ECiQ	Automatic analyzer ECiQ	Automatic analyzer ECiQ
Detection range	10% CV at 11.1pg/mL	10% CV at 11.1pg/mL	10% CV at 11.1pg/mL
Reference range	0-900pg/mL	0-900pg/mL	0-900pg/mL
Troponin I			
Analytical method	VITROS	VITROS	VITROS

	Immunodiagnostic Products Troponin I ES Reagent Pack	Immunodiagnostic Products Troponin I ES Reagent Pack	Immunodiagnostic Products Troponin I ES Reagent Pack
Equipment	Automatic analyzer ECiQ	Automatic analyzer ECiQ	Automatic analyzer ECiQ
Detection range	0.012-80ng/mL	0.012-80ng/mL	0.012-80ng/mL
Reference range	0-0.034ng/mL	0-0.034ng/mL	0-0.034ng/mL

Supplemental Table 2. Association between individual abnormal markers (D-dimer, N-Terminal Pro-Brain Natriuretic Peptide, and troponin I) and acute pulmonary embolism prognosis.

**Markers**	**Hazard ratio (95% CI)**
D-dimer	4.49 (0.57-24.19)
NT-Pro BNP	2.17 (1.12-8.19)
cTnI	2.74 (1.45-5.16)
D-dimer + NT-Pro BNP	3.90 (2.00-15.61)
D-dimer + cTnI	2.69 (1.20-5.58)
NT-Pro BNP + cTnI	3.06 (1.07-8.89)

Adjusted for age, sex, heart rate, SBP, smoking, alcohol consumption, change in psychological status, SaO2, disease history, treatment, and prescription adherence for APE recurrence.
